# In Vivo Immune-Modulatory Activity of Lefamulin in an Influenza Virus A (H1N1) Infection Model in Mice

**DOI:** 10.3390/ijms25105401

**Published:** 2024-05-15

**Authors:** Susanne Paukner, Sandra Kimber, Charlotte Cumper, Tina Rea-Davies, Lorena Sueiro Ballesteros, Christopher Kirkham, Adam Hargreaves, Steven P. Gelone, Claire Richards, Wolfgang W. Wicha

**Affiliations:** 1 Nabriva Therapeutics GmbH, Leberstrasse 20, 1110 Vienna, Austria; wolfgang.wicha@nabriva.com; 2 Charles River Discovery, Portishead BS20 7AW, UK; sandy.kimber@crl.com (S.K.); charlotte.cumper@crl.com (C.C.); tina.rea-davies@crl.com (T.R.-D.); lorena.sueiroballesteros@crl.com (L.S.B.); christopher.kirkham@crl.com (C.K.); claire.richards@crl.com (C.R.); 3 PathCelerate Ltd., Cheshire CW4 8PW, UK; adam.hargreaves@pathcelerate.com; 4 Nabriva Therapeutics US, Inc., Fort Washington, PA 19034, USA

**Keywords:** acute respiratory distress syndrome (ARDS), acute lung injury (ALI), community-acquired pneumonia, lefamulin, oseltamivir, azithromycin, antiviral, anti-inflammatory, lung histopathology, cytokines

## Abstract

Lefamulin is a first-in-class systemic pleuromutilin antimicrobial and potent inhibitor of bacterial translation, and the most recent novel antimicrobial approved for the treatment of community-acquired pneumonia (CAP). It exhibits potent antibacterial activity against the most prevalent bacterial pathogens that cause typical and atypical pneumonia and other infectious diseases. Early studies indicate additional anti-inflammatory activity. In this study, we further investigated the immune-modulatory activity of lefamulin in the influenza A/H1N1 acute respiratory distress syndrome (ARDS) model in BALB/c mice. Comparators included azithromycin, an anti-inflammatory antimicrobial, and the antiviral oseltamivir. Lefamulin significantly decreased the total immune cell infiltration, specifically the neutrophils, inflammatory monocytes, CD4^+^ and CD8^+^ T-cells, NK cells, and B-cells into the lung by Day 6 at both doses tested compared to the untreated vehicle control group (placebo), whereas azithromycin and oseltamivir did not significantly affect the total immune cell counts at the tested dosing regimens. Bronchioalveolar lavage fluid concentrations of pro-inflammatory cytokines and chemokines including TNF-α, IL-6, IL-12p70, IL-17A, IFN-γ, and GM-CSF were significantly reduced, and MCP-1 concentrations were lowered (not significantly) by lefamulin at the clinically relevant ‘low’ dose on Day 3 when the viral load peaked. Similar effects were also observed for oseltamivir and azithromycin. Lefamulin also decreased the viral load (TCID_50_) by half a log10 by Day 6 and showed positive effects on the gross lung pathology and survival. Oseltamivir and lefamulin were efficacious in the suppression of the development of influenza-induced bronchi-interstitial pneumonia, whereas azithromycin did not show reduced pathology at the tested treatment regimen. The observed anti-inflammatory and immune-modulatory activity of lefamulin at the tested treatment regimens highlights a promising secondary pharmacological property of lefamulin. While these results require confirmation in a clinical trial, they indicate that lefamulin may provide an immune-modulatory activity beyond its proven potent antibacterial activity. This additional activity may benefit CAP patients and potentially prevent acute lung injury (ALI) and ARDS.

## 1. Introduction

Community-acquired pneumonia (CAP) is often accompanied by strong pro-inflammatory responses mediated by macrophages and monocytes [1]. IL-6 and TNF-α are the major pro-inflammatory cytokines in the early innate immune responses to both bacterial and viral infections [1,2]. For bacterial CAP cases, IL-6 and TNF-α serum levels have been directly correlated with early death independent from the bacterial pathogen [3]. Similarly, hyperinflammation and macrophage activation due to influenza virus H1N1 or SARS-CoV-2 can result in an overwhelming immune response characterized by the overproduction of pro-inflammatory cytokines, such as IL-1ß, IL-6, TNF-α, and IFN-γ [4,5,6,7]. This outcome is also referred to as the ‘cytokine storm’, which is directly correlated to tissue injury, an unfavorable prognosis and usually accompanied by high mortality rates [2,7]. Acute lung injury (ALI) and respiratory distress syndrome (ARDS) may be the worst forms of complication that are characterized by a dysregulated inflammation, inappropriate accumulation and activity of leukocytes and platelets, uncontrolled activation of coagulation pathways, and altered permeability of alveolar endothelial and epithelial barriers leading to cyanosis, hypoxemia, pulmonary edema, and increasing respiratory failure over time, resulting in multiorgan failure and a high mortality rate of up to 60% [4,7,8,9]. Therefore, controlling the dysregulated inflammation and alveolar infiltration by leukocytes is considered an important cornerstone in the therapy of severe CAP besides the mandatory elimination of the causative microorganisms. The concept of immunomodulatory therapy in viral infections has also been successfully used in the most recent SARS-CoV-2 pandemic for the treatment of severe COVID-19 to impede an overwhelming immune response [10,11,12]. For the treatment of bacterial pneumonia, the focus of therapy has mainly been on the reduction in the bacterial burden by the mandatory use of effective antibiotics [13]. However, the inclusion of adjunctive immunomodulators in basic therapy has gained more interest, and the clinical effectiveness of the adjunctive use of immunomodulators or antimicrobials with immune-modulatory properties has been demonstrated [12,14,15,16]. The reduction in the pro-inflammatory cytokines IL-6, IL-1ß, and TNF-α appears to be particularly important since persistent cytokine elevation has been correlated with mortality [17,18,19]. Although neutrophils and monocytes, which are recruited through the exposure to these cytokines and chemokines, are important to control bacterial pneumonia by killing the bacteria through phagocytosis or DNA-based neutrophil extracellular traps, an overwhelming activation and failure to de-escalate the immune response has been linked with ARDS [15,20]. Both, antibacterials with anti-inflammatory activity and antivirals plus anti-inflammatory agents (e.g., corticosteroids) have been established as immunomodulatory CAP therapies [2,21,22].

The Immunomodulatory potential of antibacterial agents and resulting therapeutic applications are widely acknowledged for various antimicrobial drug classes including macrolides, tetracyclines, aminoglycosides, sulfonamides, sulfones, and others [12,23]. Particularly, azithromycin is appreciated by clinicians for its additional immunomodulatory properties in the treatment of CAP and chronic inflammatory diseases of the respiratory tract (e.g., bronchiectasis, diffuse panbronchiolitis, cystic fibrosis, chronic obstructive pulmonary disease, chronic rhinosinusitis, asthma) and other sites of infections (e.g., blepharitis, periodontitis) [12,24,25]. These include a decrease in the number of neutrophils, decreased concentrations of interleukins (IL-8, IL-6, IL-1β), tumor necrosis factor α (TNF-α), eosinophilic cationic protein, and matrix metalloproteinase (MMP)-9, as well as an inhibition of neutrophil function and decreased T helper (Th)2 cell cytokines (IL-4, IL-5, IL-6) and less frequently, Th1 cytokines (IL-2, INF-γ) [24,26].

Lefamulin is the first-in-class systemic pleuromutilin antimicrobial approved for the treatment of CAP in adults [27,28] and displays potent antibacterial activity and clinical efficacy against the most common typical and atypical bacterial CAP pathogens [29,30,31,32,33,34,35] and pathogens causing other infectious diseases [36,37]. In addition to its potent antibacterial activity, earlier studies indicated additional anti-inflammatory activity, but knowledge about the anti-inflammatory and immunomodulatory properties of this compound class and particularly lefamulin is still limited [38,39,40]. The anti-inflammatory activity of lefamulin was recently demonstrated in a lipopolysaccharide (LPS)-induced lung neutrophilia model in mice, in which the levels of pro-inflammatory cytokines (TNF-α, IL-6, IL-1β, and GM-CSF), chemokines (CXCL-1, CXCL-2, and CCL-2), and MMP-9 were significantly and dose-dependently reduced in mouse lung tissue with lefamulin [39]. Similarly, valnemulin, a pleuromutilin derivative used in veterinary medicine for the treatment of infections in swine and poultry for >15 years, has been reported to display anti-inflammatory in vitro and in vivo activity in a lipopolysaccharide (LPS)-induced acute lung injury model in mice [38,40].

The goal of this study was to evaluate lefamulin’s anti-inflammatory and immunomodulatory properties in the murine ARDS model induced by the influenza virus A/H1N1, a model that is able to replicate most clinical and pathological changes observed in human ARDS. The antiviral comparator oseltamivir and the anti-inflammatory antimicrobial azithromycin served as positive controls.

## 2. Results

### 2.1. Influenza A/H1N1 Infection and Treatment Schedule

All animals were infected intranasally at Day 0 with the mouse-adapted influenza virus strain A/Puerto Rico/8/34 (A/PR/8/34, H1N1) at ~100 TCID_50_ units (corresponding to 70 plaque-forming units (PFUs)). Infected and untreated mice showed typical symptoms of severe pneumonia and ARDS characterized by 80% lethality on Day 6 post-inoculation, highly edematous lungs, inflammatory cellular infiltration, alveolar and interstitial edema, lung hemorrhage, hypoxemia, elevated levels of pro-inflammatory cytokines and chemokines, and significant body weight loss.

Treatment started on Day −1 for all treatment groups (pretreatment) and included a vehicle control group (placebo, water for injection (WFI) BID 0 mg/kg SC), two dosing regimens for lefamulin, one azithromycin, and one oseltamivir treatment group that were treated for a total duration of 8 days (by Day 6, Table 1). The ‘low’ dose regimen for lefamulin (Dose 1) was subcutaneously (SC) administered lefamulin at 35 mg/kg twice daily (BID; daily dose 70 mg/kg). The ‘high’ dose lefamulin regimen (Dose 2) started with the SC administration of 35 mg/kg TID (three times daily). Since the animals showed signs of distress including piloerection and body weight loss, where the animals started to present behavior anticipating the next dose, the three-times-a-day treatment was reduced. The dosing regimen of Dose 2 was changed to a BID administration of 70 mg/kg lefamulin, thereby increasing the daily dose from 105 mg/kg to 140 mg/kg on Days 3 to 6 (Table 1) and in doing so reduced the handling of severely sick animals. Similarly, azithromycin treatment was started on Day −1 with SC 15 mg/kg BID but was altered in life. The azithromycin-treated animals presented aversive behavior following the SC treatment, and therefore the route of administration was switched to intraperitoneal (IP) dosing on Days 1 to 6 according to internal Charles River Laboratories (CRL) guidelines. Oseltamivir was orally administered at 20 mg/kg/day once daily (SID) from Days −1 to 6 with no dose adjustments required.

### 2.2. Survival, Body Weight, Lung Weight, and Lung Consolidation

**Figure 1:**
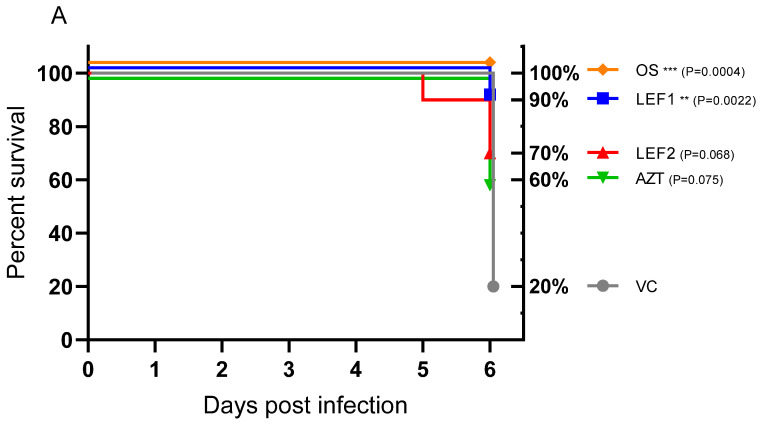

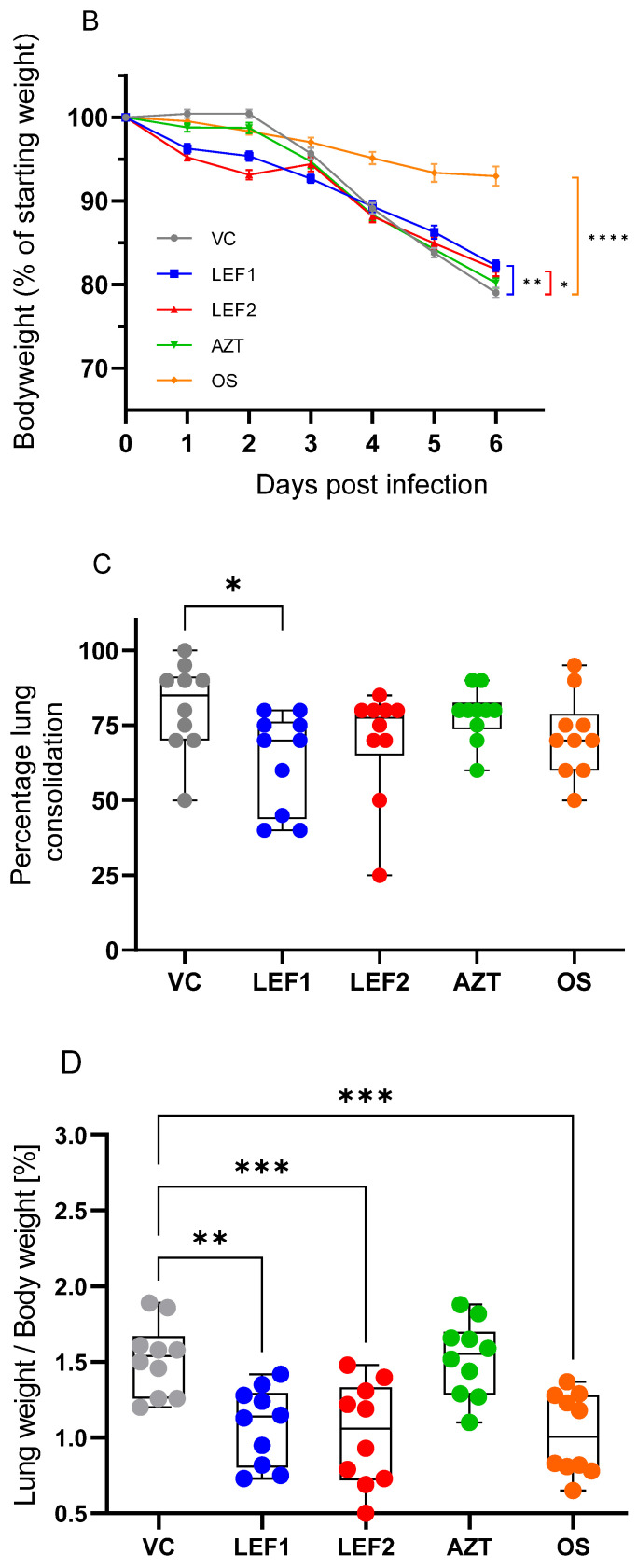
Effect of lefamulin, oseltamivir, and azithromycin treatment on the survival, body weight, lung consolidation, and wet lung weight of mice infected with influenza virus A (H1N1). BALB/c mice were infected intranasally at Day 0 with the mouse-adapted influenza virus strain A/Puerto Rico/8/34 (A/PR/8/34, H1N1). Treatment started at Day −1 (pretreatment) and included a ‘low’ dosing regimen for lefamulin (LEF 1, BID 35 mg/kg SC until Day 6 p.i.), a ‘high’ dosing regimen for lefamulin (LEF 2, TID 35 mg/kg SC until Day 2 p.i. and switched to BID 70 mg/kg SC until Day 6 p.i.), azithromycin (AZT, BID 15 mg/kg SC until Day 0 p.i. and switched to BID 15 mg/kg IP until Day 6 p.i.), oseltamivir (OS, QD 20 mg/kg PO until Day 6 p.i.), and the vehicle control (VC, BID WFI, 0 mg/kg SC). (**A**) Survival rate monitored daily. Data are presented as a Kaplan–Meier survival plot with percent survival plotted against time (*n* = 15 to Day 3, *n* = 10 thereafter). Day 6 data analyzed by log-rank (Mantel–Cox) test, comparing against the vehicle group. (**B**) Body weight monitored daily. Data are presented as mean percentage body weight loss compared to that on Day 0 (pre-infection) ± SEM (*n* = 15 to Day 3, *n* = 10 thereafter). All groups demonstrated significant body weight losses on Day 6 when compared to Day 0 (not indicated in the graph). Day 6 data analyzed by one-way ANOVA followed by Dunnett’s multiple comparisons test, comparing against the vehicle group. (**C**) Lung consolidation of mice at Day 6 p.i. Data are presented as box–whisker plots showing individual data points, median, and minimum and maximum of lung consolidation at Day 6 (*n* = 10). Data analyzed by one-way ANOVA followed by Dunnett’s multiple comparisons test, comparing against the vehicle group. (**D**) Lung wet weight per body weight of mice (%) at Day 6 p.i. Data are presented as box–whisker plots showing individual data points, median, and minimum and maximum of wet lung weight/body weight at Day 6 (*n* = 10). Data analyzed by one-way ANOVA followed by Dunnett’s multiple comparisons test, comparing against the vehicle group. Significance shown where *, **, ***, and **** represent significance levels of *p* ≤ 0.05, *p* ≤ 0.01, *p* ≤ 0.001, and *p* ≤ 0.0001, respectively.

Eight of the ten (80%) untreated vehicle group animals had reached their clinical endpoint by Day 6 (based on body weight loss compared to their start weight), i.e., two (20%) of the vehicle group animals survived. In the lefamulin groups regimen 1 and 2, survival was 9 of 10 (90%) and 7 of 10 (70%), respectively. All animals that received oseltamivir survived to the end of the study, but only 6 of 10 (60%) azithromycin-treated animals (Figure 1A). Vehicle-treated animals started to lose body weight on Day 3 and had an average of 79% of their initial body weight by Day 6 (Figure 1B), which is typical for this model [41,42]. Local intolerances appeared to be the cause of early body weight losses and higher (not significant) clinical scores in the initial two days for lefamulin regimens 1 and 2 above those typically observed in the influenza model and therefore not observed in the vehicle group. This outcome was considered to be linked with the distress that was observed for the TID dosing regimen of lefamulin (TID 35 mg/kg SC) on Days −1 to 2. Upon adaptation of the dosing regimen to the BID dosing of lefamulin (BID 70 mg/kg SC) on Day 3, the body weight loss curve flattened. Azithromycin-treated groups showed body weight curves similar to the vehicle, whereas oseltamivir demonstrated the least body weight loss with a mean AUC of % body weight loss of 20.92% per day.

At termination on Day 6, the lungs of all the animals (*n* = 10 per group) were assessed for gross pathology using a semi-quantitative scale also referred to as lung consolidation. All treatment groups showed a lower level of lung consolidation when compared to the vehicle-treated control (Figure 1C). The ‘low dose’ lefamulin (dosing 1, LEF 1) was the only group for which the reduction in lung consolidation was statistically significant. Accumulation of pulmonary edema was quantitatively measured using gravimetric means, by the determination of the wet lung weight on Day 6 for each individual animal corrected by the individual body weights. When compared with the vehicle control, oseltamivir and both lefamulin dosing regimens showed significantly reduced wet lung weight/body weight ratios, while there was no significant difference between azithromycin and the vehicle control (Figure 1D).

### 2.3. Lung Viral Titer

**Figure 2:**
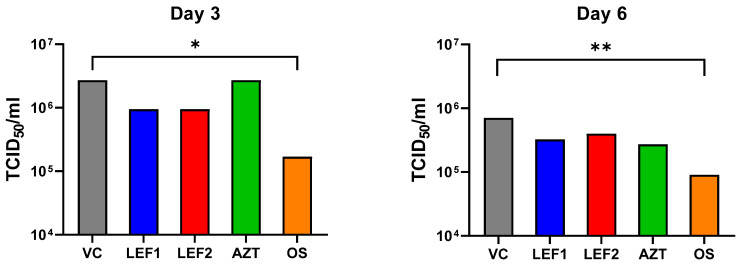
Lung viral titer. TCID_50_ value from animals at Day 3 and 6 (*n* = 5 on Day 3, *n* = 10 on Day 6). Each lung sample was serially diluted and the cytopathic effect on MDCK cells assessed in singlicates. The dilutions at which 50% of the samples were infected were used to calculate one group TCID_50_. Based on infective dose on MDCK cells. Data analyzed as a survival curve by log-rank Mantel–Cox test where * indicates *p* ≤ 0.05, ** indicates *p* ≤ 0.01. N.B., the analysis generated a single value per group. Individual TCID_50_ values were not generated for each animal.

Lung viral titers were assessed by visually scoring the cytopathic effect (CPE) on Madin-Darby canine kidney (MDCK) cells to enumerate the quantity of virus in the lung homogenates. Viral titers peaked on Day 3 and slightly declined on Day 6 in the untreated vehicle control and the treatment groups (Figure 2 and Supplementary Material Table S1). Animals receiving oseltamivir treatment had a significantly reduced viral titer (median tissue culture infectious dose, TCID_50_) of approximately −1 log_10_ compared to the vehicle-treated control at both timepoints. A small, not significant reduction in the lung viral titer of half a log10 was observed for both lefamulin treatment regimens, at Day 3 and Day 6, when compared to the vehicle-treated control. A similar, not significant reduction in the viral titer when compared to the vehicle control was also observed for azithromycin on Day 6, while the virus titer on Day 3 was the same as for the vehicle control (Figure 2).

### 2.4. Immune Cell Infiltration in the Lung

**Figure 3:**
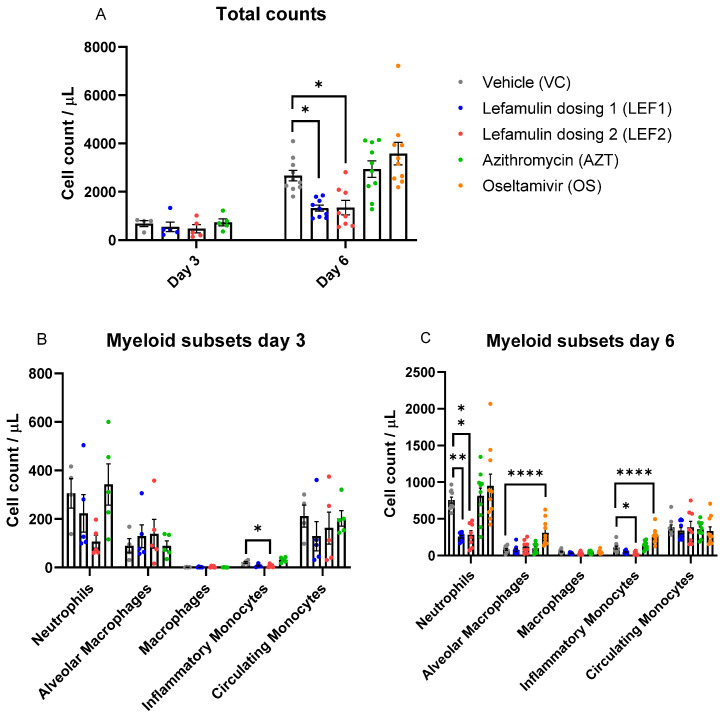

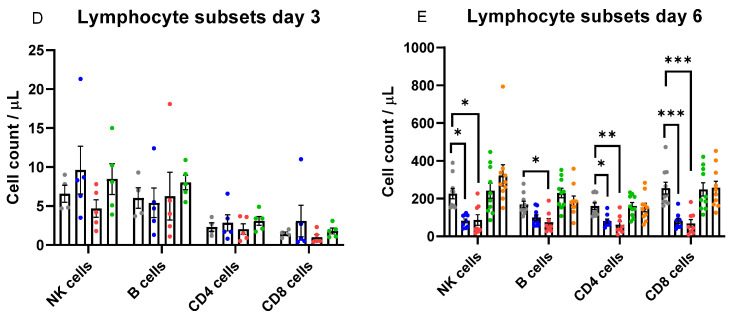
Immune cell infiltration into the lung after influenza virus A (H1N1) challenge in animals receiving different treatments. (**A**) Total counts, (**B**,**C**) myeloid subsets, and (**D**,**E**) lymphocyte subsets in the BALF on Day 3 (*n* = 5 animals per treatment group) and Day 6 (*n* = 10 animals per treatment group). Oseltamivir was not assessed on Day 3 due to a technical issue. Bars represent mean ± SEM. One animal from Day 3 vehicle control, one animal from Day 6 lefamulin test dose 1, and two animals from Day 6 lefamulin test dose 2 were excluded from the charts for being outliers (see raw data files in Supplementary Material Table S3). Ordinary one-way ANOVA and Dunnett’s multiple comparisons against the vehicle control treatment were run for each timepoint. *, **, ***, and **** represent significance levels of *p* ≤ 0.05, *p* ≤ 0.01, *p* ≤ 0.001, and *p* ≤ 0.0001, respectively.

In the untreated vehicle control, the total immune cell counts in the bronchoalveolar lavage fluid (BALF) obtained by flow cytometry increased by Day 6 when compared with the naïve animals as expected. Lefamulin significantly decreased the total immune cell infiltration into the lung by Day 6 at both doses tested when compared with the vehicle control, while that of azithromycin and oseltamivir was similar to the vehicle control (Figure 3A). Among the myeloid subset, lefamulin specifically reduced the inflammatory monocyte and neutrophil counts at Day 3 and more markedly on Day 6 when compared with the vehicle control. Both changes were significant for lefamulin dosing 2 on Day 6, while only the reduction in neutrophils by lefamulin dosing 1 was significant on Day 6. The cell counts on Days 3 and 6 in the azithromycin group were similar to those of the vehicle control for all myeloid cells. Though some azithromycin-treated animals demonstrated low neutrophil counts on Day 6, the difference was statistically not significant compared to the vehicle control (Figure 3B,C). In the oseltamivir group, the neutrophil counts were similar to those of the vehicle control and the inflammatory monocytes were significantly higher than the vehicle control on Day 6. Day 3 samples for oseltamivir could not be assessed because of a technical issue and reference is made to an earlier study [43]. The number of alveolar macrophages remained constant on Days 3 and 6 for both lefamulin groups and azithromycin, while it was significantly higher for the oseltamivir group on Day 6 than for the vehicle group. The number of macrophages and circulating monocytes increased over time for all groups and treatment groups were similar to the vehicle control group.

The lymphocyte subset infiltration was similarly low for all groups on Day 3 and increased by Day 6 (Figure 3D). No substantial differences compared to the vehicle control group were observed on Day 3 for all treatment groups. At Day 6 (Figure 3E), lefamulin decreased natural killer (NK) cell, CD4^+,^ and CD8^+^ T-cell infiltration in the lung in a dose-dependent manner. This reduction was significant when compared to the vehicle control for all cells, with the exception of the B-cell count at lefamulin dose 1. In contrast, the lymphocyte subset infiltration for azithromycin and oseltamivir on Day 6 was similar to the vehicle control group.

### 2.5. Cytokines and Chemokine Concentrations in BALF and Plasma

**Figure 4:**
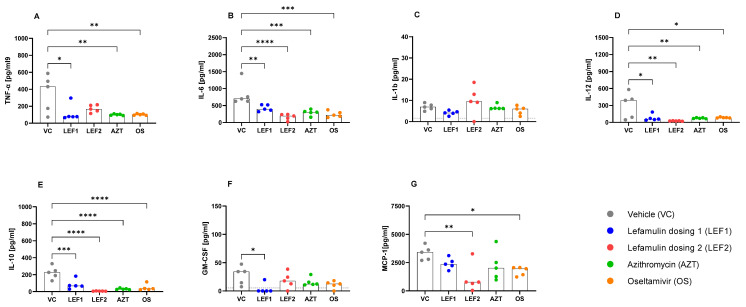
BALF concentrations of cytokines primarily produced by monocytes and macrophages on Day 3, when the virus load peaked. Data show concentrations of TNF-α (**A**), IL-6 (**B**), IL-1ß (**C**), IL-12p70 (**D**), IL-10 (**E**), and growth factors GM-CSF (**F**), MCP-1 (**G**). Each data point represents an animal, with the bar showing the median value across all animals within the group (*n* = 5 animals per treatment group). N.B., values below the Lower Limit of Quantitation (LLOQ) are plotted as zero. Statistical significance between the vehicle control and treatment groups were determined using one-way ANOVA with Dunnett’s post-hoc test. *, **, ***, and **** represent significance levels of *p* ≤ 0.05, *p* ≤ 0.01, *p* ≤ 0.001, and *p* ≤ 0.0001, respectively. Dotted grey line represents LLOQ.

**Figure 5:**
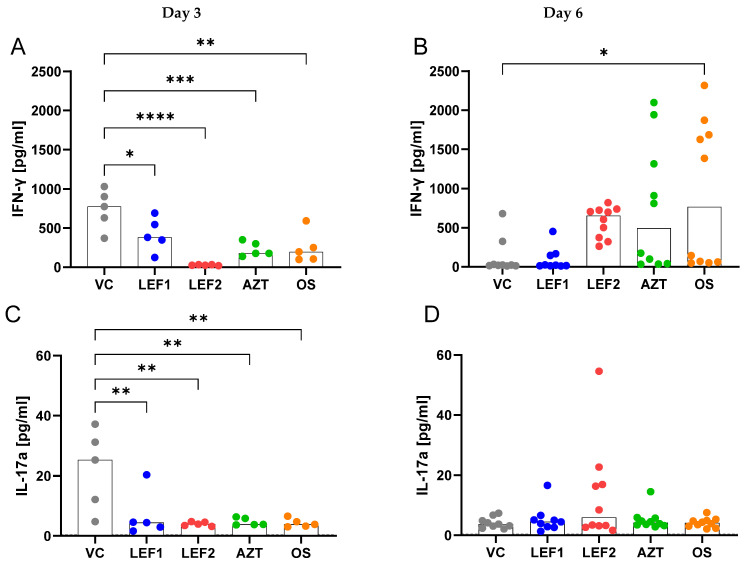

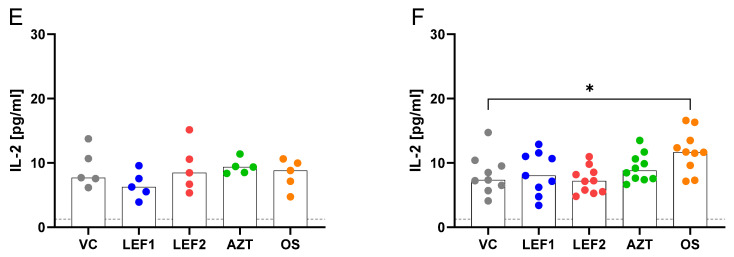
CD4^+^ T-cell-related cytokine concentrations in BALF on Days 3 and 6. Data show concentrations of IFN-γ (**A**,**B**), IL-17a (**C**,**D**), and IL-2 (**E**,**F**) on Day 3 (*n* = 5 animals per treatment group) and Day 6 (*n* = 10 animals per treatment group). Each data point represents an animal, with the bar showing the median value across all animals within the group. N.B., values below the Lower Limit of Quantitation (LLOQ) are plotted as zero. Statistical significance between the vehicle condition and treatment groups, and vehicle and control groups was determined using one-way ANOVA with Dunnett’s post-hoc test. *, **, ***, and **** represent significance levels of *p* ≤ 0.05, *p* ≤ 0.01, *p* ≤ 0.001, and *p* ≤ 0.0001, respectively. Dotted grey line represents LLOQ.

Most of the measured cytokine, chemokine, and growth factor concentrations (IL-1β, IL-2, IL-6 IL-10, IL-12p70, IL-17a, IFN-γ, TNF-α, GM-CSF, MCP-1) of the vehicle control were more elevated in the BALF than in the plasma on Day 3 post-infection (Figure 4 and Figure 5 and Figure S1 in the Supplementary Material), when the viral titers peaked (Figure 2) and as seen in other studies using this model (data on file at CRL).

In comparison to the vehicle, lefamulin (‘low’ dose) resulted in significant decreases in IL-6, TNF-α, IL-10, IL-12p70, and GM-CSF in the BALF and slight, not significant decreases in IL-1ß and MCP-1 on Day 3, all cytokines/chemokines and growth factors that are primarily produced by monocytes and macrophages (Figure 4). Among the cytokines that are primarily produced by CD4^+^ T-cells, the IFN-γ and IL-17a concentrations were significantly decreased by the ‘low’ dose lefamulin on Day 3, while IL-2 was unaffected (Figure 5). Lefamulin (‘high’ dose) resulted in more pronounced and significant decreases in IL-6, IL-10, IL-12p70, MCP-1, IFN-γ, and IL-17a, and not-significant decreases in TNF-α and GM-CSF. No significant changes were observed for IL-1ß and IL-2 (Figure 4 and Figure 5). Oseltamivir demonstrated significant decreases in IL-6, IL-10, IL-12p70, IL-17a, IFN-γ, MCP-1, and TNF-α, and not-significant decreases in GM-CSF compared to the vehicle group on Day 3, while IL-1β and IL-2 were similar to the vehicle control. In the azithromycin group, a significant decrease was seen on Day 3 for IL-6, TNF-α, IL-12p70, IL-10, IL-17a, and IFN-γ) and a not-significant decrease for GM-CSF and MCP-1, while IL-1β and IL-2 were unchanged.

On Day 6 (Supplementary Material, Figure S1), some pro-inflammatory cytokine levels in the BALF increased for oseltamivir, azithromycin, and lefamulin when compared to the vehicle control, e.g., IL-1β, IL-2, IFN-γ for oseltamivir and azithromycin, and IL-6 and IL-12p70 for the ‘high’ dose lefamulin, which may reflect an altered progression of infection and cytokine production kinetics. The Day 6 BALF levels of TNF-α, IL-17A, GM-CSF, and MCP-1, for oseltamivir, azithromycin, and lefamulin doses 1 and 2, were low and not significantly different than those of the vehicle control. The anti-inflammatory cytokine IL-10 in the BALF was significantly increased for the lefamulin ‘high’ dose 2, but not the low dose, and not significantly increased for oseltamivir and azithromycin.

Where cytokines were increased in the plasma (Supplementary Material, Figure S1) at Day 3 in the vehicle-treated group (IL-2, IL-12p70, IFN-γ), a reduction was observed with the oseltamivir and azithromycin treatment, but not with lefamulin at both doses. However, at Day 6, with the plasma cytokine levels in the vehicle-treated animals back at basal levels, the reverse was true for azithromycin and oseltamivir. Cytokine increases were observed for IL-2, IL-10, IL-12p70, IL-17A, IFN-γ, and TNF-α for the azithromycin and oseltamivir treatment groups, with slightly higher levels observed after oseltamivir treatment. GM-CSF significantly increased at Day 6 for oseltamivir only, which appears to be linked with monocyte infiltration in the lung. Treatment with lefamulin at both doses showed no significant effects on the plasma cytokine levels at Day 3 compared to the vehicle-treated control. A significant increase in IFN-γ, with smaller but not significant increases in IL-2, IL-10, IL-12p70, and IL-17A on Day 6, was seen in the group treated with the ‘high’ lefamulin dose (but not for the ‘low’ lefamulin dose), which is consistent with an increased clinical score in this group and distress related to the drug administration.

### 2.6. Histopathology

**Figure 6:**
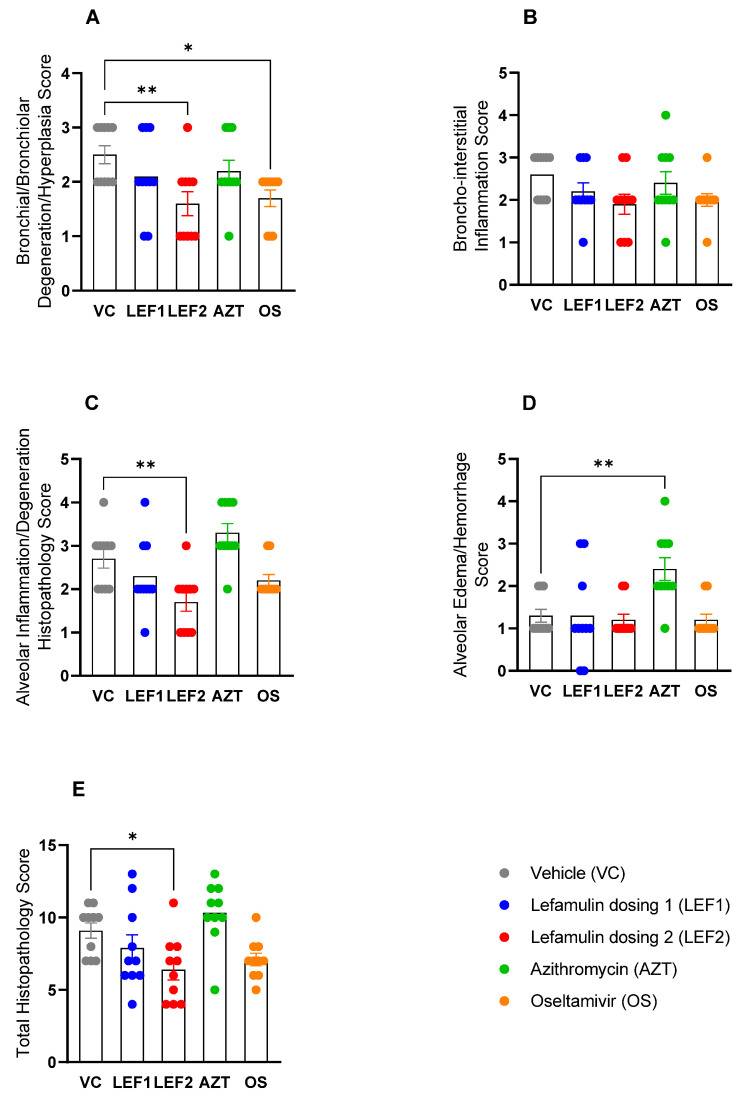
Histopathological scores on Day 6 p.i. (**A**) Bronchial/bronchiolar degeneration/hyperplasia, (**B**) broncho-interstitial inflammation, (**C**) alveolar inflammation/degeneration histopathology, (**D**) alveolar edema/hemorrhage, and (**E**) total histopathology scores. Data are presented as mean histopathology score + SEM (*n* = 10). Each sample is scored out of a maximum score of 4 (criteria, see Supplemental Material, Table S1). Data analyzed by one-way ANOVA followed by Dunnett’s multiple comparisons test, comparing against the vehicle group. Significance shown where * indicates *p* ≤ 0.05 and ** indicates *p* ≤ 0.01.

**Figure 7:**
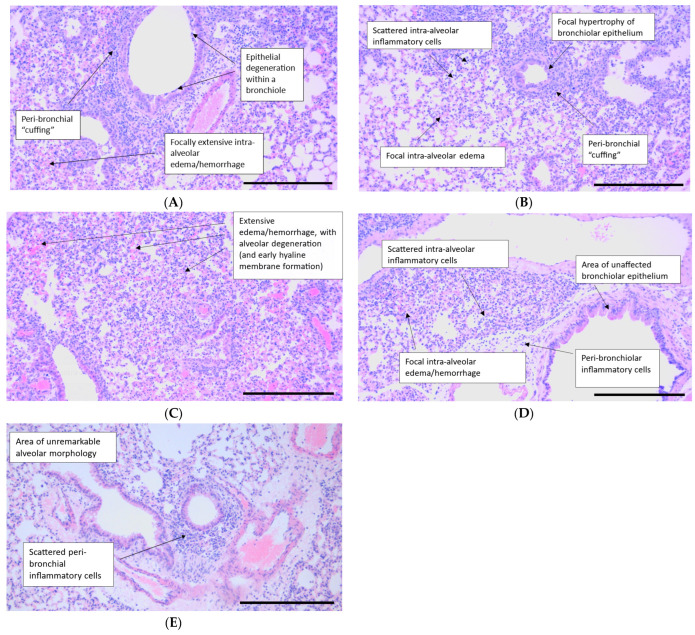
Histological images of representative lung slides for all treatment groups. Lung tissue injury was assessed by hematoxylin and eosin staining on Day 6 p.i. Original magnification: ×100. Scale bar = 500 µm. (**A**) Vehicle control animal showing focally extensive intra-alveolar hemorrhage/edema; extensive peri-bronchial inflammation (“cuffing”); and notable bronchial epithelial degeneration. (**B**) Oseltamivir-administered animal showing a reduction in bronchiolar degeneration, when compared to (**A**). (**C**) Azithromycin-administered animal demonstrating marked alveolar hemorrhage/edema, correlating with increases in group organ weight. (**D**) Lefamulin (dosing 1)-administered animal that shows changes predominantly within the alveolar field. (**E**) Lefamulin (dosing 2)-administered animal that shows a reduction in bronchiolar degeneration and alveolar inflammation/degeneration within this field of view, when compared to (**A**).

Histopathological assessment of hematoxylin and eosin-stained lung sections was graded semi-quantitatively in a blinded manner. Histopathology endpoints across the entire study were varied with a total score range of 4–13 evident within the specimens examined (Figure 6). Lesions of the vehicle control (Figure 7A) were similar to those described within the literature, with H1N1 A/PR8/34 influenza virus, in BALB/c mice [44,45,46]. Increasing severities/distributions of alveolar pathology generally were consistent with increases in bronchiolar degeneration/proliferation, broncho-interstitial inflammation, and alveolar edema/hemorrhage, signifying stepwise global parenchymal involvement (Figure 6A–E and Figure 7A).

Lefamulin treatment resulted in a dose-dependent, and for the ‘high’ lefamulin dose 2 significant, reduction in bronchial degeneration (Figure 6A) and alveolar inflammation (Figure 6C), with a concomitant decrease in broncho-interstitial inflammation (not significant, Figure 6B). This resulted in an overall significant reduction in the histopathology score for this group, when compared to the vehicle-treated control group (Figure 6E). Representative histological images for both lefamulin dose groups are shown in Figure 7D,E. In contrast, azithromycin did not result in significantly different histopathology scores than the vehicle group, even significantly increasing the alveolar edema/hemorrhage histopathology score (Figure 6D, histological image in Figure 7C), and being consistent with increases in the lung weight (Figure 1D). Oseltamivir significantly reduced the bronchial/bronchiolar degeneration/hyperplasia histopathology score (Figure 6A, representative histological image in Figure 7B) while the other scores and the total histopathology score were similar to those of the vehicle control group (Figure 6B–E).

## 3. Discussion

This study investigated the anti-inflammatory activity of lefamulin, a potent antibacterial agent, in an ARDS model induced by influenza virus A H1N1. By infecting mice with the mouse-adapted influenza virus H1N1 A/PR8/34, it is possible to assess the antiviral efficacy in vivo together with assessing the ability of immunomodulators to control viral-driven immunopathology and cytokine production [21,44,47,48,49]. The influenza infection in BALB/c mice progressed as expected with the development of severe pneumonia, a significant reduction in clinical scores, significant body weight loss, and a mortality rate of 80% in the vehicle control (=placebo) group. Pneumonia was accompanied by the development of pulmonary edema, lung infiltration by leukocytes, the production of pro-inflammatory cytokines and chemokines in the BALF and plasma, and elevated histopathological scores, bronchial/bronchiolar degeneration/hyperplasia, broncho-interstitial inflammation, alveolar inflammation/degeneration, and alveolar edema/hemorrhage. All were similar to those described in the literature for this influenza virus strain in BALB/c mice [44,45,46] and for ARDS in mice [49].

Two dosing regimens were evaluated for lefamulin in the study: a ‘low’ dose (dosing 1; BID 35 mg/kg SC) and a ‘high’ dose (dosing 2; TID 35 mg/kg SC switched to BID 70 mg/kg SC). The 35 mg/kg BID SC dose (daily dose of 70 mg/kg/day) in mice is exposure equivalent to the intravenous and oral dosage forms of lefamulin approved for the treatment of CAP in adults (BID 150 mg IV or BID 600 mg oral) [27,50]. The ‘high’ lefamulin dosing 2 was 105 and 140 mg/kg/day, respectively, in order to enhance the likelihood of achieving statistically significant results when compared to the ‘low’ dose and to the vehicle control. Dosing regimen 2 started with a TID administration of 35 mg/kg SC but had to be adjusted to BID dosing (of a higher dose) since the drug administration three times a day caused too much additional distress to the mice, which started to present behavior anticipating the next dose. Azithromycin at a daily dose of 30 mg/kg SC and IP in mice slightly exceeds the exposure following a 500 mg oral dose in humans already on Day 1 (assuming linear pharmacokinetics) [51,52]. The change in the route of administration from SC to IP had to be performed according to the internal animal welfare guidelines, since the severely sick animals were distressed by the SC dosing of azithromycin. IP dosing improved the burden on the animals, and is not considered to affect the drug exposure based on the good relative bioavailability [53]. Oseltamivir and the tested dosing regimen of QD 20mg/kg/day PO was included as the gold-standard antiviral positive control that was optimized in this model to reduce lung pathology and increase survival.

Lefamulin, oseltamivir, and azithromycin had a positive outcome on survival and lung consolidation across the study. Notably, a reduction in lung consolidation was only significant for the ‘low’ lefamulin dose (dosing 1). Though lung consolidation was also reduced for the ‘high’ dose lefamulin, this was not significant. Lefamulin and oseltamivir further significantly reduced the wet lung weights/body weights by Day 6 (Figure 1D), indicating the attenuation of pulmonary edema by these treatments. Although this easy gravimetric method has some limitations and does not allow discrimination between hydrostatic and permeability edema, the data are considered useful and indicative for edema formation and the evaluation of lung injury [54].

Lung viral titers were lower on Day 6 than on Day 3 in all groups. This is in line with the expected outcomes in other influenza studies whereby the lung viral load peaks on Day 3 (CRL, data on file). Oseltamivir resulted in significantly reduced lung viral titers on Days 3 and 6 when compared with the vehicle-treated control group. Unexpectedly, both lefamulin doses also showed virus titers of approximately half a log_10_ lower than the vehicle control, but these differences were not statistically significant and there was no dose–response correlation. The reported antiviral activity of azithromycin [55] did not translate into any measurable effect in viral titers at the tested dosing regimen at Day 3, whereas a TCID_50_ reduction by approximately half a log_10_ was measured on Day 6.

In the same period, immune cell infiltration increased markedly in the vehicle control. Treatment with the anti-inflammatory drug azithromycin did not alter immune cell infiltration compared to the vehicle control. The viral inhibitor oseltamivir caused increases in the recruitment of inflammatory monocytes by Day 6 and led to a higher count of alveolar macrophages, which is indicative of reduced lung damage [56]. This effect could be due to a change in the viral pathogenesis time course, resulting in a peak closer to Day 6 than in the other groups. Treatment with lefamulin significantly decreased the total immune cell infiltration in the lung by Day 6 at both doses tested, while that of the anti-inflammatory antibiotic azithromycin and the antiviral oseltamivir groups was similarly high as the vehicle control. By Day 6, both lefamulin doses significantly decreased neutrophils, inflammatory monocytes (only lefamulin dose 2 significant), natural killer cell, CD4^+^ and CD8^+^ T-cell infiltration into the lung, and also reduced B-cells, which was only significant for LEF regimen 2. Neutrophils are the first immune cells to migrate to the lungs during immune infections and are crucial for removing virus particles, infected and dying cells, the recruitment of more neutrophils, activating T-cell responses, and honing B-cell activity. But on the other hand, unmitigated neutrophil-mediated activity in the alveolar space can cause hyperinflammation and acute tissue injury [57]. Therefore, the reduction in neutrophils along with the reduction in inflammatory monocytes appears to be important in the attenuation of an overwhelming innate immune response and is in line with the observed attenuation of effects on the adaptive immune response. If the observed effects on T- and B-cells may result in a potentially reduced protective immunity against influenza virus or if the effects endorse the early clearance of the viral infection are unknown and require further examination. However, the observed effects on cell infiltration into the BALF are in line with the observed histopathology (Figure 6 and Figure 7), where lefamulin treatment resulted in a dose-dependent reduction in bronchial degeneration, alveolar inflammation, and total histopathology score. A reduction (not significant) in the histopathological score was also observed for oseltamivir as expected [43] but not for azithromycin at the tested dosing regimen that was exposure equivalent to the human clinical dose but lower than doses used in earlier in vivo studies [55,58].

Furthermore, the cellular infiltration and histopathology results are consistent with the cytokine levels in the BALF at Day 3, which proved more informative than the levels in the plasma or Day 6 cytokine concentrations in the BALF. At Day 3, when the viral load was at its highest, the lefamulin, azithromycin, and oseltamivir treatments resulted in reduced pro-inflammatory cytokines. In some cases, the relative levels were reversed by Day 6 in the azithromycin-, oseltamivir-, and the ‘high’ dose lefamulin-treated groups when compared with the vehicle-treated control. The timepoint taken to screen cytokines is critical. A possible explanation for the reversal is that the peak of cytokine production had passed by Day 6 in the vehicle group, whereas in the lefamulin, azithromycin, and oseltamivir groups, the treatment might have altered the kinetics of disease progression, thus shifting the peak closer to Day 6.

Both lefamulin doses resulted in significant decreases in the early pro-inflammatory cytokines IL-6 and TNF-α, which play a central role in the innate immune response and a potential ‘cytokine storm’. Uncontrolled TNF-α production leads to the excessive activation of NF-κB, MAPK, and STAT3 signaling pathways, followed by the excessive production of cytokines and chemokines, forming a cytokine storm. TNF-α can also damage the endothelial barrier, and cause pulmonary edema and tissue damage [4]. Similarly, IL-6 plays an important role in the cytokine storm via the JAK2/STAT3 pathway in viral and bacterial pneumonia and is a predictive marker of inflammation indicative for a poor prognosis of influenza patients [4]. Lefamulin also decreased the IL-12 levels in the BALF, which is involved in the differentiation of naïve T-cells into Th1 cells and the enhancement in the cytotoxic activity of NK cells and CD8^+^ cytotoxic T-cells. This is in line with the observed attenuation of lung infiltration by these lymphocytes (Figure 3). ‘Low’ lefamulin (dosing 1) further significantly decreased the Day 3 BALF concentrations of the granulocyte-macrophage colony-stimulating factor (GM-CSF) but not the Day 6 BALF concentrations. GM-CSF is an immune-modulating cytokine that plays a critical role in maintaining the alveolar epithelium and pulmonary immune system under homeostatic and pathologic conditions, including infection [59,60]. Inhibition by lefamulin as early as on Day 3 (but not on Day 6) correlates with reduced IL-6 and TNF-α levels and seems to be related to the reduction in influenza progression and pro-inflammatory response. Since no GM-CSF inhibition was observed on Day 6, lefamulin might not negatively affect any beneficial effects GM-CSF may have in lung repair in viral infections. Monocyte chemoattractant protein-1 (MCP-1/CCL2) is one of the key chemokines that regulate the migration and infiltration of monocytes/macrophages. Inhibition by lefamulin at both doses on Day 3 corresponded with the reduced number of inflammatory monocytes in the BALF on Day 3. Also in line with histopathology are the concentrations of IL-10, which is known as an anti-inflammatory mediator capable of down-regulating acute lung inflammation, but also with an ambiguous role, since elevated plasma levels have been correlated with the accelerated mortality of lung contusion and pneumonia in mice [61] and higher mortality of ARDS and CAP patients [62,63]. The IL-10 levels in the BALF were significantly lower than those of the vehicle control for all test compounds on Day 3, but increased by Day 6 in the BALF and also in the plasma for oseltamivir, azithromycin, and the ‘high’ lefamulin dose (Figure 4 and Figure S1).

Among the CD4^+^ T-cell-related cytokines, lefamulin at both doses significantly reduced the interferon-γ (IFN-γ) and IL-17a concentrations in the BALF on Day 3, as was observed for azithromycin and oseltamivir (Figure 5). On Day 6, the INF-γ BALF concentrations went down to normal in the vehicle group and the lefamulin dose 1 group, while the IFN-γ levels remained elevated for lefamulin 2 (not significant), azithromycin (not significant), and oseltamivir (significant). None of the dosing regimens affected the IL-2 concentrations in the lung on Days 3 and 6 with the exception of oseltamivir, which increased the IL-2 concentrations significantly on Day 6. Dysregulated interferon signaling, including excessive IFN-γ production primarily by T-cells, promotes lung injury and impairs lung repair during influenza pathogenesis, and IFN-γ exerts a direct pathologic effect by promoting epithelial injury, resulting in the exacerbation of pulmonary inflammation and loss of barrier integrity during influenza [16]. Moreover, IFN-γ has been proposed as an intriguing target of immunotherapies for influenza-complicated bacterial pneumonia (secondary bacterial pneumonia), because influenza-induced IFN-γ drives inflammatory hypercytokinemia and ultimately lethal lung damage during secondary MRSA pneumonia, despite antibiotic therapy [64]. IL-17 links T-cell activation to neutrophil mobilization and activation and as such can mediate protective immunity, but on the other hand can in combination with TNF-α also contribute to the pathogenesis of inflammatory and autoimmune diseases, such as psoriasis, rheumatoid arthritis, or periodontitis. Moreover, it was shown that influenza virus-induced lung damage in mice could be ameliorated by the neutralization of IL-17, and that IL-17 may play a pathogenic role in lung inflammation and in ARDS associated with COVID-19 [65].

The reduction in IL-6, TNF-α, INF-γ, IL-12p70, MCP-1, IL-17a, and IL-10 lung concentrations in this ARDS model by lefamulin are consistent with those observed in the LPS-induced lung neutrophilia mouse model where single SC doses of 35, 70, and 140 mg/kg lefamulin resulted in reduced cytokine and chemokine concentrations (TNF-α, IL-6, IL-1β, and GM-CSF, CXCL-1, CXCL-2, CCL-2, MMP-9) in lung tissue and reduced infiltration of the lung by inflammatory monocytes and neutrophils when compared to the vehicle control [39].

Overall, treatment with the antiviral oseltamivir (QD 20 mg/kg PO) appeared efficacious in the inhibition of influenza virus replication early on, and in the suppression of the development of influenza virus-induced bronchi-interstitial pneumonia in the majority of structural endpoints as expected. Treatment with the anti-inflammatory antimicrobial azithromycin at the tested dose (BID 30 mg/kg SC and IP) resulted in slightly reduced viral titers at the end of the study, and reduced BALF concentrations of pro-inflammatory cytokines as reported in earlier publications. However, azithromycin did not show reduced pathology in terms of lung consolidation, pulmonary edema/hemorrhage formation, histopathology, and pulmonary immune cell infiltration. This is possibly due to the severity of this influenza virus model, in which the successful alleviation of acute pulmonary injury required significantly more efficacious therapeutic intervention at higher azithromycin doses. The results further indicate that lefamulin at both doses, at the ‘low’ dose (dosing 1, BID 35 mg/kg SC) that is pharmacokinetically equivalent to the clinical dosing but more notably at the ‘high’ dose (dosing 2, TID 35 mg/kg SC and BID 70 mg/kg SC), modulates the innate immune response in terms of reduced pulmonary lung infiltration by neutrophils and inflammatory macrophages, reduced production of pro-inflammatory cytokines, and attenuated lung pathology associated with viral infection. But this study also had some limitations. One limitation is the administration adaptation during dosing, since the azithromycin IP dosing and the SC lefamulin TID dosing were not well tolerated initially by the influenza virus-infected mice. Adapted dosing regimens (SC administration of azithromycin and BID dosing of the ‘high’ lefamulin) improved the overall tolerance until the end of the study but could not outweigh the clinical scores and body weight losses due to the dosing in the first few days. Since lefamulin is approved for CAP via both an intravenous and an oral dosage form, it would be worth further investigating the immunomodulatory potential after oral administration in this model. Furthermore, anti-inflammatory drugs, e.g., dexamethasone, which was used in the COVID-19 pandemic for the treatment and prevention of a ‘cytokine’ storm, and/or liposomal dexamethasone formulations [21], should be included as a comparator in a follow-up study. The measurement of an even broader panel of cytokines, chemokines, and growth factors at various timepoints may also be advisable for the comprehensive evaluation of lefamulin’s immunomodulatory activity. While this and previous studies only describe the immune-modulatory effects of lefamulin in in vivo models, more work is required to investigate the mode of action on a molecular biological level as well as in viral and bacterial pneumonia in vivo models.

## 4. Materials and Methods

### 4.1. Test Compounds

Lefamulin acetate salt (batch 17NB26.HE00011) was provided by Nabriva Therapeutics (AT) and was stored at room temperature. Azithromycin (batch 19114) was supplied by Aspire Pharma (Petersfield, UK) and oseltamivir (batch F01156) was purchased from Santa Cruz Biotechnology (Dallas, TX, USA). Test drug stock solutions of lefamulin (7 mg/mL in WFI), azithromycin (3 mg/mL in WFI), and oseltamivir (4 mg/mL in WFI) were prepared fresh each day and stored at 2–8 °C upon use.

### 4.2. ARDS Model—Experimental Outline and Viral Infection

Adult 8-week-old female BALB/c mice were challenged with intranasal administration of 35μL of the mouse-adapted influenza virus strain A/Puerto Rico/8/34 (A/PR8/34, H1N1) on Day 0. Allantoic fluid was diluted 1/50,000× corresponding to approximately 100 TCID_50_ units or 70 plaque-forming units per mouse [44]. From Day 0 until the end of the experiment, animals were weighed daily and were scored daily for clinical signs of influenza virus infection (=clinical score) to include abnormal coat condition (piloerection), abnormal posture (hunched), abnormal breathing (rapid and/or irregular breathing rate), reduced mobility, ocular discharged, and/or eye closure.

The group size was *n* = 15 animals per treatment group and included *n* = 5 satellite animals for Day 3 analyses and *n* = 10 animals for Day 6 analyses. At Day 3, five satellite animals per group were terminated, lungs were weighed, and processed for viral titer determination. At Day 3 and at the end of the experiment on Day 6, terminal blood from 5 and 10 animals, respectively, was collected and processed into plasma for cytokine quantification by centrifugation at 4 °C. BALF samples were also collected and processed for cytokine analysis (multiplex technology) and flow cytometry analysis of immune cell components. At termination on Day 6, lungs were assessed for gross pathology using a semi-quantitative scale also referred to as lung consolidation. A small lobe was preserved in fixative (10% Formalin Cellstor, CellPath, Powys, UK) and stored for histopathology. The remaining lung samples were stored at −80 °C and processed at a later date to determine the Day 6 viral titer.

### 4.3. Treatment

Treatment with test drugs started on Day −1 (pre-treatment) at clinically relevant doses (lefamulin 70 mg/kg/day and 105/140 mg/kg/day, subcutaneous (SC), azithromycin 30 mg/kg/day, SC and intraperitoneal (IP), and oseltamivir 20 mg/kg/day per os (PO)) and continued to Day 6 according to the dosing regimens presented in Table 1. BALF was collected on Days 3 and 6 to measure infiltrating lung leukocytes, cytokines, and chemokines. Lung immunopathology following infection was evaluated on Day 6 by gross pathology at termination together with hematoxylin and eosin histopathology.

### 4.4. Quantification of Viral Load (TCID_50_ Assay)

On Days 3 and 6, lungs were collected, homogenized, and clarified to determine viral load by TCID_50_ assay on Madin-Darby canine kidney (MDCK) cells. The TCID_50_ assay is used to quantify viral titers by determining the concentration at which 50% of the infected cells display a cytopathic effect (CPE). Each lung sample was serially diluted, and a sample of each dilution added onto the cell monolayers. The samples were incubated for 3 days at 35 °C/5%CO_2_, in a humidified atmosphere. Following incubation, the cell monolayers were visually scored for cytopathic effect (cell rounding) indicating the presence of influenza virus. At least three diluted samples were required to generate the TCID_50_ titer; therefore, all the singlicate samples from one group were used to generate a group TCID_50_ value. Analysis followed the method determined earlier [66].

### 4.5. Flow Cytometry Analysis of BALF

BALF samples were collected, and cells processed for flow cytometry analysis using the following antibody panel by following the manufacturer’s instructions: CD45, TCRβ, CD3, CD4, CD8, CD19, Ly6C, Ly6G, MHCII, CD11b, CD11c, CD49b, Siglec-F, CD64, and eFluor™ 780 viability dye. Cells were fixed post-staining in 1% paraformaldehyde then analyzed the following day using the BD LSR-Fortessa-X20 cytometer. Absolute cell counts were performed using flow cytometry CountBright™ Absolute Counting Beads (ThermoFisher, Waltham, MA, USA) by following the manufacturer’s instructions. Data were analyzed using FlowJo v10.7 and graphed using GraphPad Prism v8. Samples were gated on size and granularity FSC v SSC > single cells > live CD45^+^. To identify neutrophils, cells were gated on Ly6G^+^. To identify alveolar macrophages, cells were gated on Ly6G^−^ > CD11b^+^ Ly6C^−^ > CD64^+^ Siglec-F^+^. To identify macrophages, cells were gated on Ly6G^−^ > CD11b^+^ Ly6C^−^ > CD64^+^ Siglec-F^−^. To identify circulating monocytes, cells were gated on Ly6G^−^ > CD11b^+^ Ly6C^+^ > MHCII^−^. To identify inflammatory monocytes, cells were gated on Ly6G^−^ > CD11b^+^ Ly6C^+^ > CD64^+^ MHCII^+^. To identify CD4^+^ and CD8^+^ cells, cells were gated on Ly6G^−^ > CD11b^−^ Ly6C^−^ > TCRβ^+^ > CD4^+^ or CD8^+^. To identify NK cells, cells were gated on Ly6G^−^ > CD11b^int^ > SSC^low^>Ly6C^−^ > CD64^−^ SiglecF^−^. To identify B-cells, cells were gated on Ly6G^−^ > CD11b^−^ Ly6C^−^ > TCRβ^−^ > CD19^+^ (gating strategy shown in Supplementary Material, Figure S2).

### 4.6. Cytokine Analysis (BALF and Plasma)

At Days 3 and 6, blood was collected and processed to isolate plasma; bronchoalveolar lavage (BAL) samples were collected and processed. Cytokine content in both types of samples was analyzed using the Bio-Plex Pro Mouse Cytokine, Chemokine, and Growth Factor Assays and the Bio-Plex 200 system (Bio-Rad Laboratories, Watford, UK) by following the manufacturer’s instructions with no modifications. Reference standards were included in duplicate for every analyte analyzed, in order to quantify cytokine and chemokine. Results from samples were interpolated into standard curves for each analyte. Standard diluent was used as a blank. The analytes quantified were as follows: IL-1β, IL-2, IL-6, IL-10, IL-12p70, IL-17, IFN-γ, GM-CSF, MCP-1, and TNF-α.

### 4.7. Statistics

All data are expressed as mean ± standard deviation, except the histopathology score. Differences between groups were examined for statistical significance using GraphPad Prism v8. Specific information about the statistical test used is provided in the respective figure legends.

### 4.8. Histopathology

Lungs from 10 animals per group at the Day 6 termination were dissected out and inflated/preserved in formalin for 24–48 h, before paraffin embedding, sectioning, and processing to glass slides, at a thickness of 4–5 µm. Histopathological assessment of the hematoxylin and eosin-stained slides was performed by a board-certified pathologist using light microscopy [44,45,46]. The histopathologist was blinded to the treatment groups to avoid observer bias, and the whole section was graded semi-quantitatively to a specific set of morphologic criteria (each with a maximum score of 4; Supplemental Material, Table S2).

## 5. Conclusions

By evaluating this data set, it can be concluded that lefamulin at both doses, but more notably at the ‘high’ dose, successfully suppresses the development of broncho-interstitial pneumonia induced by the instillation of A/PuertoRico/8 (H1N1) (PR8) in BALB/c mice. Lefamulin improved the survival rates, and attenuated lung consolidation, pulmonary edema formation, and histopathology. Furthermore, lefamulin demonstrated anti-inflammatory/immunomodulatory activity by reducing the BALF levels of pro-inflammatory cytokines and growth factors including IL-6, TNF-α, IFN-γ, IL-12p70, IL-17a, IL-2, GM-CSF, and MCP-1. The effects were consistent with the reduced lung infiltration by neutrophils, inflammatory monocytes, CD4^+^ and CD8^+^ T-cells, and NK cells. These results underscore the potential of lefamulin beyond its known potent antibacterial activity to prevent a ‘cytokine storm’ and subsequent ALI/ARDS in patients with severe viral pneumonia. This beneficial effect may be of particular importance for those CAP patients where the causative agent (viral vs. bacterial aetiology) is unknown or when a secondary bacterial pneumonia is suspected in addition to a viral pneumonia and requiring antibacterial treatment. Moreover, the results from this study warrant additional studies to evaluate the immunomodulatory potential of lefamulin and its relevance in the treatment of pneumonia, the prevention and/or treatment of ARDS, and the management of chronic inflammatory diseases.

## Figures and Tables

**Table 1:** Treatment groups and dosing regimen.

Group	Group Size (N)	Dose and Regimen	Daily Dose (mg/kg)	Treatment Days
Vehicle control (WFI)	15	BID 0 mg/kg SC	0	Days −1 to 6
Lefamulin (Regimen 1)	15	BID 35 mg/kg SC	70	Days −1 to 6
Lefamulin (Regimen 2)	15	TID 35 mg/kg SC	105	Days −1 to 2
		for 4 days followed by		
		BID 70 mg/kg SC ^a^	140	Days 3 to 6
Azithromycin	15	BID 15 mg/kg SC	30	Days −1 to 0
		BID 15 mg/kg IP ^b^	30	Days 1 to 6
Oseltamivir	15	QD 20 mg/kg PO	20	Days −1 to 6

Abbreviations: BID, twice daily; IP, intraperitoneal; QD, once daily; PO, oral; SC, subcutaneous; TID, three times per day; WFI, water for injection. ^a^ Dosing regimen was changed post-Day 2 from TID dosing to BID dosing, since animals were distressed by the TID administration. ^b^ SC azithromycin dosing was changed to IP dosing on Day 1 for better tolerability.

## Data Availability

Data are available at Nabriva Therapeutics GmbH, Vienna, Austria and at Charles River Laboratories, Portishead, United Kingdom.

## Supplementary Materials

The following supporting information can be downloaded at: https://www.mdpi.com/article/10.3390/ijms25105401/s1.
