# Supplementary material for: In Vivo Immune-Modulatory Activity of Lefamulin in an Influenza Virus A (H1N1) Infection Model in Mice
Source: Int J Mol Sci. 2024 May 15;25(10):5401. doi: 10.3390/ijms25105401 (PMC11121702; doi:10.3390/ijms25105401)
Supplement: Supplementary file 1 [file ijms-25-05401-s001.zip › ijms-2979014-supplementary.pdf]

Supplementary Material to

In Vivo Immune-Modulatory Activity of Lefamulin in an Influenza Virus A (H1N1) Infection Model in Mice

Susanne Paukner, [1] Sandra Kimber [2], Charlotte Cumper [2] , Tina Rea Davies [2], Lorena Sueiro Ballesteros [2], Christopher Kirkham [2], Adam Hargreaves[4], Steven P. Gelone [3], Claire Richards [2] and Wolfgang W. Wicha [1]

Table of Contents

Figure S1. Cytokine, chemokine and growth factor concentrations in plasma and BALF on Days 3 and 6.

Table S1. Lung Viral Titers on Day 3 and Day 6.

Table S2. Histopathology Score Criteria.

Figure S2. Flow cytometric gating strategy for immune cells in BALF.

Table S3. Raw data for the analysis of immune cell infiltration into the lung.

Figure S1. Cytokine, chemokine and growth factor concentrations in (A) plasma and (B) BALF on Days 3 and 6. Each data point represents each animal, with the bar showing the median value across all animals within the group. N.B. Values below the Lower Limit of Quantitation (LLOQ) are plotted as zero. Statistical significance between the vehicle condition and treatment groups, and vehicle and control groups were determined using One-way ANOVA with Dunnett's post-hoc test. * - p≤ 0.05, ** - p≤0.005, *** - p≤0.0005, **** - p≤0.0001. Dotted grey line represents LLOQ.

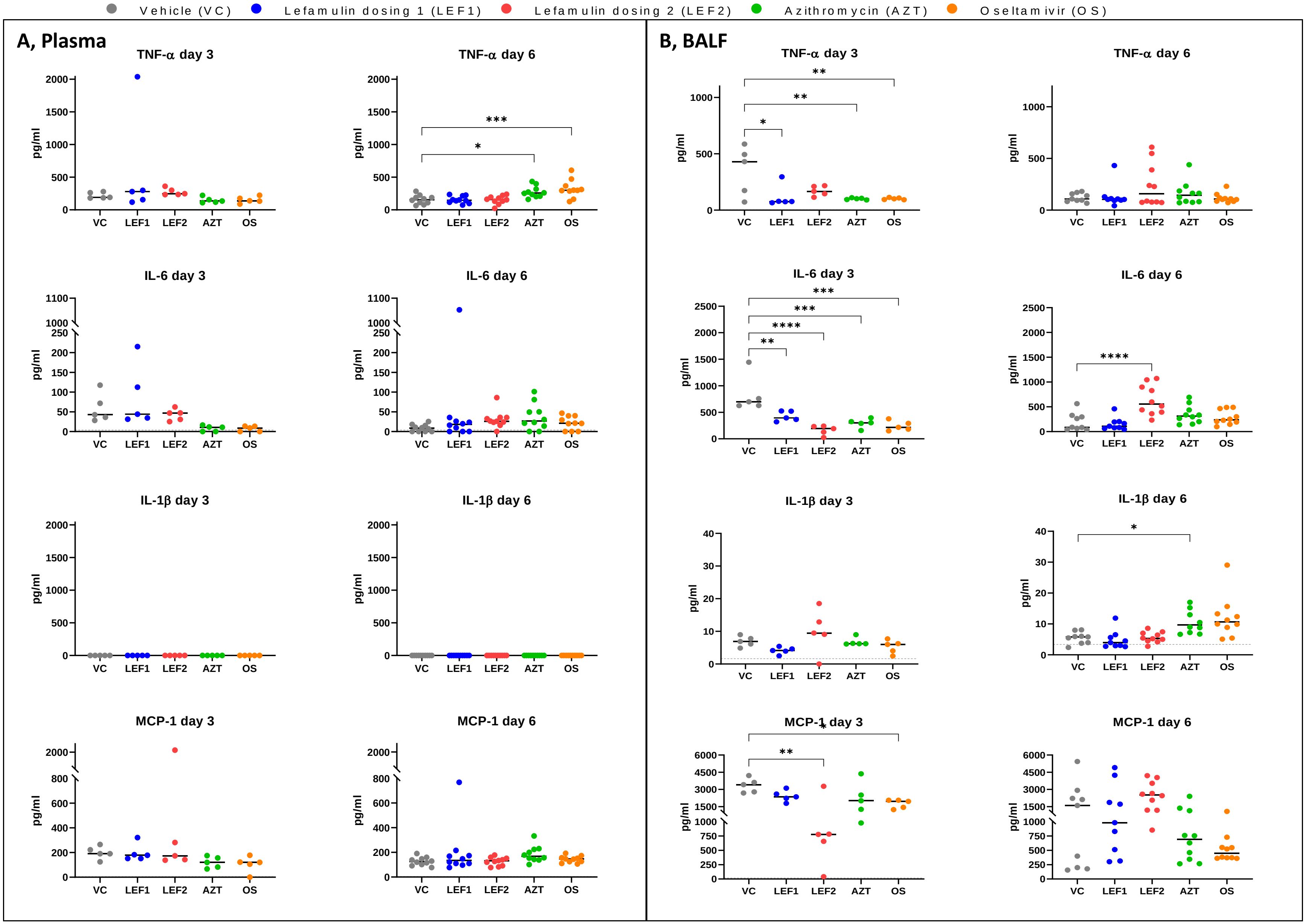

Figure S1. Continued.

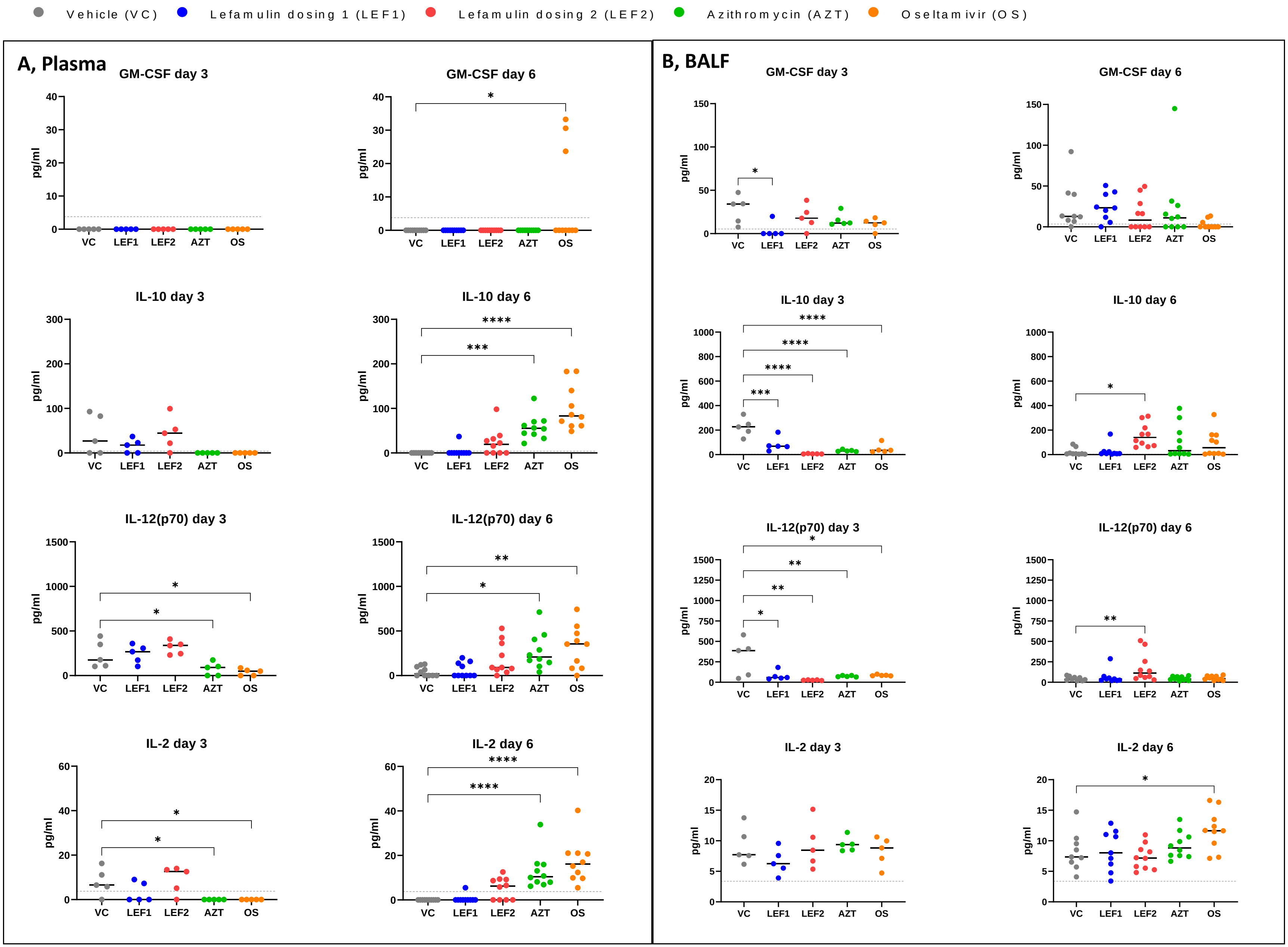

Figure S1. Continued.

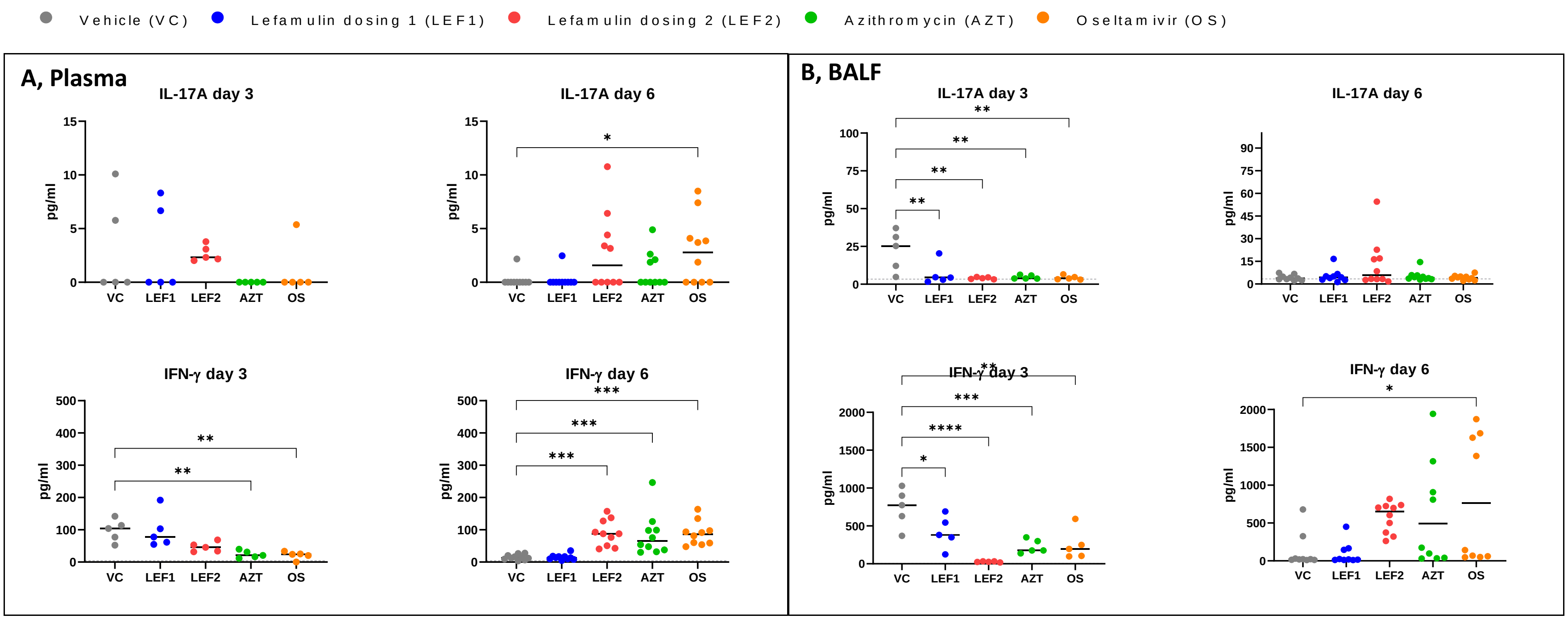

Table S1. Lung Viral Titer. $TCID_{50}$ value from animals at Day 3 and 6 (Day 3 n=5, Day 6 n=10). Based on infective dose on MDCK cells.

**Group**	**Day 3 $TCID_{50}$/mL**	**Day 6 $TCID_{50}$/mL**
Vehicle	2.72E+06	7.05E+05
Lefamulin Reg. 1	0.95E+06	4.00E+05
Lefamulin Reg. 2	0.95E+06	3.24E+05
Azithromycin	2.72E+06	2.72E+05
Oseltamivir	0.17E+06	0.90E+05

Table S2. Histopathology Score Criteria. Lungs at the Day 6 termination from 10 animals per group were dissected out and preserved in formalin. Samples were prepared into slides for histopathological assessment with haematoxylin and eosin stains. The histopathologist was blinded to the treatment groups. Each sample was scored by the following criteria:

Bronchial/Bronchiolar Degeneration/Hyperplasia

0 - Unremarkable

1 - Focal degeneration/necrosis of epithelial cells

2 - Focal degeneration/necrosis of epithelial cells; hypertrophy of epithelial cells

3 - Multi-focal degeneration/necrosis of epithelial cells; mild hyperplasia of epithelial cells

4 - Multi-focal degeneration/necrosis of epithelial cells; moderate hyperplasia of epithelial cells

Broncho-interstitial Inflammation

0 - Unremarkable

1 - Scattered subacute mixed inflammatory cells (neutrophils, admixed with lymphocytes and mononuclear cells) surrounding bronchi/bronchioles

2 - Partial (discontinuous) “cuffing” of airways by mixed inflammatory cells

3 - Circumferential (complete/confluent) “cuffing” of airways by mixed inflammatory cells

4 - Circumferential (complete/confluent) “cuffing” of airways by mixed inflammatory cells; expansion into interstitial septae

Alveolar Inflammation/Degeneration

0 - Unremarkable

1 - <20% field infiltration (across all lobes) by mixed inflammatory cells

2 - 20 - <50% field infiltration by mixed inflammatory cells; focal areas of septal degeneration/necrosis

3 - 50 - <75% field infiltration by mixed inflammatory cells; multi-focal areas of septal degeneration/necrosis

4 - >75% field infiltration by mixed inflammatory cells; confluent areas of septal degeneration/necrosis

Alveolar Edema/Haemorrhage

0 - Unremarkable

1 - focal, discreet, areas of intra-alveolar edema/haemorrhage

2 - multi-focal, discreet, areas of intra-alveolar edema/haemorrhage

3 - confluent areas of intra-alveolar edema/haemorrhage; local vascular degeneration

4 - >50% (across all lobes) of field occupied by intra-alveolar edema/haemorrhage; extensive vascular degeneration

Figure S2. Flow cytometric gating strategy for immune cells in BALF. On day 0, animals were challenged with H1N1 influenza by intranasal administration. Animals were then treated with test and control treatments based on the administration schedule. Bronchoalveolar lavage was performed on each animal, and the BAL fluid processed for the cellular infiltrate. The cells were stained with a viability dye and a cocktail of antibodies. Counting beads were added before acquisition for cell enumeration. All samples were analysed on the BD Fortessa X20. Further analysis was performed on the FlowJo software. Total cells were gated on scatter > single cells > live CD45+. Subsets were gated on scatter > single cells > live CD45+. To identify neutrophils, cells were gated on Ly6G+. To identify alveolar macrophages, cells were gated on Ly6G- > CD11b+ Ly6C- > CD64+ Siglec-F+. To identify macrophages, cells were gated on Ly6G- > CD11b+ Ly6C- > CD64+ Siglec-F-. To identify circulating monocytes, cells were gated on Ly6G- > CD11b+ Ly6C+ > MHCII-. To identify inflammatory monocytes, cells were gated on Ly6G- > CD11b+ Ly6C+ > CD64+ MHCII+. To identify CD4 and CD8 cells, cells were gated on Ly6G- > CD11b- Ly6C- > TCRβ+ > CD4+ or CD8+. To identify NK cells, cells were gated on Ly6G- > CD11bint > SSClow>Ly6C- > CD64- SiglecF-. To identify B cells, cells were gated on Ly6G- > CD11b- Ly6C- > TCRβ- > CD19+. Gating strategy is a representative sample from day 6 vehicle control group.

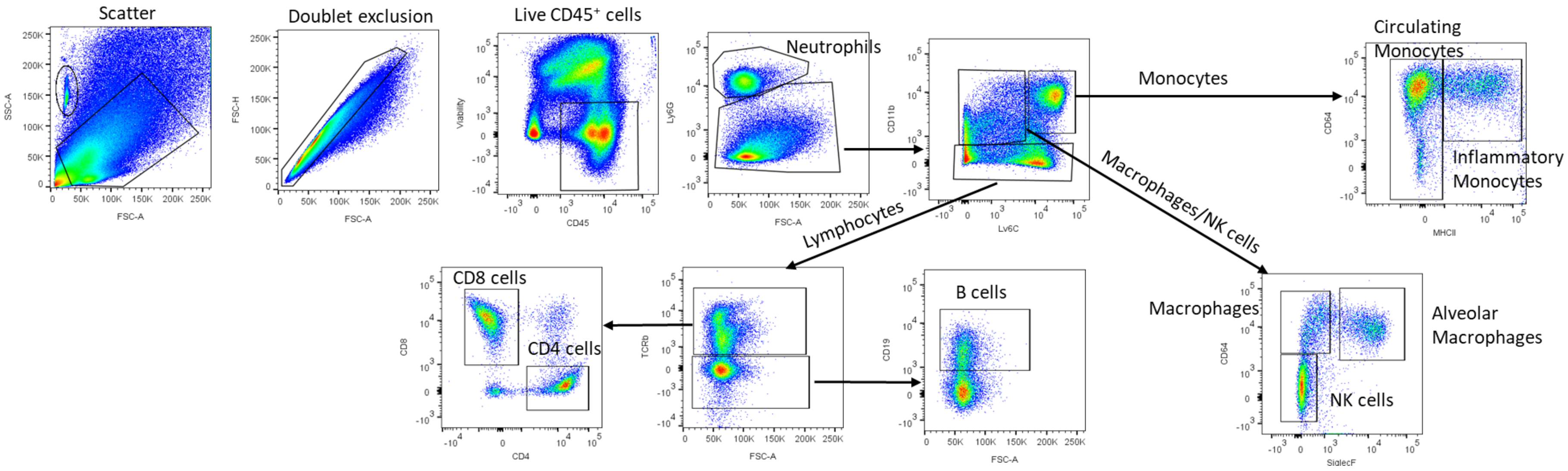

Table S3. Raw data for the analysis of immune cell infiltration into the lung.

Day 3		Cells/μL									
	**Animal ID**	**Total**	**Neutrophils**	**Alveolar Macrophages**	**Macrophages**	**Inflammatory Monocytes**	**Circulating monocytes**	**NK cells**	**B cells**	**CD4 cells**	**CD8 cells**
**Vehicle Control**	**11**	315.63	138.02	31.17	0.71	13.94	98.64	4.73	3.71	1.17	1.35
	**12**	831.97	416.39	63.01	1.20	25.18	263.89	4.77	9.27	3.60	2.03
	**13**	789.16	368.93	167.98	2.67	14.87	183.93	7.82	4.08	1.73	0.87
	**14**	789.62	300.45	95.57	1.60	30.73	301.30	9.02	6.96	2.69	1.59
	**15**	6.78#	3.55#	0.49#	0.02#	0.08#	1.15#	0.17#	0.30#	0.39#	0.10#
**Lefamulin Dose 1**	**26**	1327.80	504.15	306.13	4.49	17.82	360.72	21.34	12.39	6.59	11.05
	**27**	439.39	127.10	138.54	1.99	7.65	114.57	8.25	5.48	3.41	2.69
	**28**	215.44	105.77	56.70	0.68	1.83	31.76	3.50	1.34	0.84	0.40
	**29**	425.25	280.95	64.30	0.84	4.10	44.93	6.31	3.05	1.70	0.48
	**30**	326.95	99.54	78.45	0.95	4.94	92.65	8.72	4.68	1.70	0.82
**Lefamulin Dose 2**	**41**	685.82	197.50	155.90	6.38	9.78	254.08	6.73	5.71	3.68	2.32
	**42**	135.99	73.59	15.99	0.52	1.63	30.92	1.82	1.06	0.52	0.38
	**43**	1018.59	140.62	358.14	7.46	16.99	374.66	7.82	18.12	3.55	1.30
	**44**	308.60	62.63	90.03	1.35	4.09	113.05	4.23	3.96	1.54	0.52
	**45**	202.63	60.79	75.81	0.88	1.71	41.19	2.94	2.42	0.90	0.48
**Azithromycin**	**56**	791.94	455.28	63.01	0.52	31.08	195.15	5.12	5.45	1.69	1.07
	**57**	1224.45	600.46	138.39	1.84	42.77	321.01	14.98	10.93	4.89	3.05
	**58**	693.62	311.06	134.87	0.82	24.77	153.65	10.24	7.78	3.40	1.78
	**59**	605.78	227.77	73.01	0.74	40.24	200.95	8.09	9.13	3.45	1.95
	**60**	355.91	116.21	35.30	0.78	17.77	143.51	3.93	6.83	2.07	1.05
**Day 6**		**Cells/μL**									
	**Animal ID**	**Total**	**Neutrophils**	**Alveolar Macrophages**	**Macrophages**	**Inflammatory Monocytes**	**Circulating monocytes**	**NK cells**	**B cells**	**CD4 cells**	**CD8 cells**
**Vehicle Control**	**1**	2514.73	728.05	70.66	52.94	134.95	352.80	239.63	139.56	137.51	218.05
	**2**	2199.24	643.88	68.22	51.62	106.37	377.62	153.03	122.63	123.94	209.99
	**3**	3111.95	874.83	51.62	44.38	85.02	359.12	345.39	194.68	233.95	340.45
	**4**	4096.82	966.82	148.25	101.18	252.42	663.81	255.20	278.61	237.82	472.52
	**5**	2800.62	888.11	101.10	47.28	70.87	366.35	235.96	168.10	179.75	266.27
	**6**	1804.57	577.88	15.30	27.43	41.75	284.62	157.69	105.89	105.84	175.69
	**7**	2382.51	852.41	68.22	40.70	84.85	337.65	162.02	155.01	108.87	184.58
	**8**	2120.69	664.61	55.67	42.35	66.38	296.39	167.30	143.92	125.30	183.59
	**9**	3409.04	671.25	120.26	70.98	159.59	461.74	389.31	233.36	234.03	338.38
	**10**	2248.23	690.52	129.11	50.77	79.42	362.56	153.15	142.87	116.11	162.05

in greyed out cells indicates outliers excluded from further calculations and analyses.

	Animal ID	Total	Neutrophils	Alveolar Macrophages	Macrophages	Inflammatory Monocytes	Circulating monocytes	NK cells	B cells	CD4 cells	CD8 cells
**Lefamulin Dose 1**	**16**	8935.41#	0.00#	1854.07#	1112.44#	0.00#	454.55#	526.32#	11.96#	1100.48#	0.00#
	**17**	919.52	167.27	28.09	18.42	54.87	276.65	41.37	55.85	70.30	50.59
	**18**	1085.05	317.09	36.73	24.22	46.06	225.00	64.88	75.73	62.03	52.15
	**19**	1455.15	279.05	32.59	39.53	52.79	482.30	112.75	77.74	74.90	86.76
	**20**	1620.24	310.19	75.78	29.22	55.11	481.87	111.61	105.52	81.93	85.19
	**21**	893.79	202.20	70.34	35.43	32.59	215.17	44.20	57.37	46.48	40.44
	**22**	1853.15	245.09	41.75	43.87	81.77	492.00	123.59	168.63	150.75	173.21
	**23**	962.91	191.71	64.16	20.20	23.48	218.57	51.58	62.63	65.03	110.54
	**24**	1798.05	322.49	217.29	32.39	66.55	409.92	79.56	164.71	115.68	89.02
	**25**	1312.42	276.30	111.53	47.20	20.08	258.64	109.48	127.51	60.91	56.96
**Lefamulin Dose 2**	**31**	545.84	181.75	27.63	12.30	9.50	155.34	17.06	29.68	24.19	17.85
	**32**	2813.26	477.85	213.92	68.62	67.43	751.42	227.43	189.39	153.38	181.74
	**33**	1118.62	311.27	257.08	36.75	8.55	352.92	25.39	26.83	10.66	9.42
	**34**	1.69#	0.57#	0.20#	0.03#	0.09#	0.31#	0.06#	0.11#	0.14#	0.03#
	**35**	589.62	71.11	23.78	11.29	15.83	156.40	37.52	52.50	51.43	44.20
	**36**	2080.66	422.84	152.34	31.33	45.73	586.56	151.03	120.96	105.99	110.66
	**37**	675.77	102.39	78.14	12.13	12.45	209.24	41.56	38.56	35.09	49.69
	**38**	980.27	247.92	80.02	21.47	23.04	318.29	34.44	56.97	35.62	27.35
	**39**	1959.99	442.60	150.87	47.79	50.91	569.36	159.08	83.72	82.96	108.02
	**40**	14.73#	4.46#	0.20#	0.27#	0.55#	2.37#	1.22#	1.32#	0.60#	1.12#
**Azithromycin**	**46**	2856.96	959.18	36.77	57.43	121.18	306.71	228.67	231.00	158.16	193.14
	**47**	2354.10	551.06	36.34	38.27	160.32	326.45	157.13	236.83	144.12	210.72
	**48**	3633.00	866.11	146.81	64.46	209.01	423.19	287.69	325.29	211.52	330.74
	**49**	1494.66	387.12	54.11	14.77	57.00	213.00	136.37	107.65	93.20	146.36
	**50**	2239.50	698.66	89.47	47.71	121.32	315.10	122.76	167.60	145.68	148.31
	**51**	4120.95	1104.71	151.88	67.94	147.80	451.73	392.92	251.54	224.56	381.35
	**52**	3193.35	1343.95	157.48	62.88	86.90	374.33	189.52	171.07	112.47	184.29
	**53**	4051.91	978.70	201.73	67.39	219.42	442.56	367.59	300.64	232.78	421.39
	**54**	1274.57	253.24	18.21	17.24	87.78	189.11	86.33	152.79	79.05	115.63
	**55**	4144.27	993.03	89.07	61.02	167.38	521.34	446.91	352.20	209.74	357.42
**Oseltamivir**	**61**	3166.48	766.71	135.82	44.05	197.58	259.70	322.71	156.89	166.38	336.91
	**62**	2187.93	871.54	270.67	27.26	127.49	154.64	150.19	70.66	61.88	125.37
	**63**	3942.21	591.86	158.45	58.29	275.27	542.62	336.69	279.58	253.14	452.88
	**64**	7217.31	2068.11	541.72	102.91	496.33	707.93	793.42	359.08	283.80	401.95
	**65**	3389.98	888.82	228.89	33.14	293.70	251.88	286.82	186.51	169.10	191.34
	**66**	4344.70	1440.83	625.39	40.31	274.49	260.91	359.87	179.54	152.75	247.09
	**67**	2423.43	423.00	172.96	26.21	240.27	197.33	260.93	184.72	115.41	221.90
	**68**	3932.54	1297.95	429.71	58.83	335.16	505.82	275.33	156.54	106.56	168.94
	**69**	2550.64	503.78	166.21	43.90	189.37	168.55	240.42	185.60	143.41	269.91
	**70**	2652.41	638.25	375.40	47.66	269.45	290.59	200.12	130.44	79.20	162.11

in greyed out cells indicates outliers excluded from further calculations and analyses.
